# Green Tea Extract Reduces the Erosive Dentine Wear Caused by Energy Drinks In Vitro

**DOI:** 10.3290/j.ohpd.b2183087

**Published:** 2021-10-22

**Authors:** Blend Hamza, Sheila Antonella Paucar Rojas, Philipp Körner, Thomas Attin, Florian Just Wegehaupt

**Affiliations:** a Resident, Clinic of Orthodontics and Pediatric Dentistry, Center of Dental Medicine, University of Zurich, Zurich, Switzerland. Wrote the manuscript.; b Dental master’s student, Clinic of Conservative and Preventive Dentistry, Center of Dental Medicine, University of Zurich, Zurich, Switzerland. Performed the experiment in partial fulfilment for Master’s degree; study idea.; c Resident, Clinic of Conservative and Preventive Dentistry, Center of Dental Medicine, University of Zurich, Zurich, Switzerland. Co-wrote the manuscript.; d Professor and Clinic Director, Clinic of Conservative and Preventive Dentistry, Center of Dental Medicine, University of Zurich, Zurich, Switzerland. Performed critical evaluation of the manuscript.; e Head of the Department of Preventive Dentistry and Oral Epidemiology, Center of Dental Medicine, University of Zurich, Zurich, Switzerland. Conceived and designed the experiment and critical evaluation of the manuscript.

**Keywords:** erosive dentine wear, energy drinks, green tea, erosive potential, profilometry

## Abstract

**Purpose:**

To investigate the effect of energy drinks supplementation with green tea extract on the erosive dentine wear.

**Materials and Methods:**

Six groups of bovine dentine samples (n = 15) were subjected to four cycles erosive attacks (10 min, 25 °C) and remineralisation (artificial saliva, 60 min, 37°C) using the following formulas: tap water; green tea extract; Red Bull; Red Bull supplemented with green tea extract; Red Bull Light; Red Bull Light supplemented with green tea extract. The erosive dentine wear – ie, the irreversible dentine loss – was measured using a stylus profilometer (µm, accuracy = 40 nm).

**Results:**

Median and interquartile range (IQR) of erosive dentine wear for the tested energy drinks before and after the supplementation with green tea extract were calculated as follows: Red Bull (before: 3.3 µm (1.0)); after: 1.2 µm (0.6)); Red Bull Light (before: 3.3 µm (0.9)); after: 2.0 µm (0.4)). The difference between the groups before and after the supplementation was statistically significant (*P* <0.05). The erosive dentine wear for the tap water group was calculated at 0.4 µm (0.6) and for the green tea extract group at –1.0 µm (1.3).

**Conclusions:**

Supplementation of energy drinks with green tea extract could reduce the erosive dentine wear caused by energy drink *in vitro*.

Erosive tooth wear is an acid-related non-bacterial loss of tooth structure. Erosive tooth wear occurs when the tooth surface is exposed to an undersaturated substance with respect to tooth minerals. Unlike dental caries, erosive tooth wear does not have a specific pH value at which it occurs.^12^ When exposed to erosive substances, tooth hard tissue gets both dissolved and softened. The superficial softened parts of tooth hard tissues are susceptible to mechanical impacts and could easily be removed during toothbrushing.^2, 25^ If not controlled, erosive tooth wear could extend to dentine and start to cause patients discomfort and aesthetic complications.^20^ The interest of both patients and researchers regarding the aetiology and complications of erosive dental wear has been steadily increasing in the past years.^19^ Repeated consumption of acidic drinks (eg, soft and energy drinks, fruit juice) has been held as a predominant factor in developing erosive tooth wear.^9^ Therefore, several studies were carried out to investigate the erosive potential of acidic drinks and its modifying factors.^24, 28, 33^ The supplementation of acidic drinks with various compounds (eg, calcium, proteins, fluoride, iron) to reduce the erosive potential has been reported to be successful in previous studies.^15, 32, 35^ The protective effect of calcium and fluoride is attributed to the basic fact that a saturated/supersaturated drink – in respect to tooth minerals – would not dissolve the tooth.^35^ The protective effect of iron was attributed to the precipitation of ferric phosphate or iron oxides as a thin acid-resisting film on the tooth surface. However, a relatively high concentration of iron is needed to provide an erosive protective effect, which might lead to an unpleasant metallic taste and tooth discoloration.^32^

Also, the role of green tea as a protective agent against erosive tooth wear has already been reported.^13, 22, 26, 27^ This protective effect has been attributed to the presence of epigallocatechin-3-gallate (EGCG), which was found to inhibit matrix metalloproteinases (MMPs), and thus hamper the loss of the dentine matrix. MMPs are extracellularly zinc-dependent proteolytic enzymes that can be activated in mildly acidic conditions. When activated, MMPs have the capacity to degrade type 1 collagen in dentine.^10^ The inhibition of MMPs could be triggered in various ways (eg, altering the catalytic domain of MMPs, activate a hydrogen binding with collagenase, bind to specific sites on the enzyme).^8^ In their *in-vitro* study, Barbosa et al^5^ stated that the supplementation of soft drinks with a 1.2% green tea extract reduced their erosive potential against dentine.

Since the consumption of energy drinks in many countries is high or even considered alarming – especially among adolescents^14, 16, 31, 38^ – and since the involvement of energy drinks in the aetiology of erosive tooth wear is well-established,^11, 29, 30^ it is important to seek methods to reduce the erosive potential of these drinks. This *in-vitro* study was therefore carried out to investigate whether the supplementation of 1.2% green tea extract would reduce the erosive potential of energy drinks against dentine.

## Materials and Methods

### Sample Preparation

Ninety dentine samples were milled out of 15 bovine permanent incisors obtained from a slaughterhouse (six samples from each incisor) using a diamond trephine mill under constant water cooling (diameter = 3 mm). The samples were then embedded in acrylic resin (Paladur; Heraeus Kulzer, Hanau, Germany) and ground at 5-N force with 1200-, 2000- and 4000-grit carborundum papers (Tegramin-30, Struers, Copenhagen, Denmark) under constant water cooling for 5, 10 and 30 sec, respectively. The samples were then divided to six groups. Each group consisted of 15 dentine samples derived from 15 different incisors. Two parallel lines were scratched into the surface of each sample (totally in the embedding material and as near to dentine surface as possible) using a sharp metal pen fixed on custom-made device to help as a reference for the profilometric analysis. Before the erosion attacks, reference areas perpendicular to the above-mentioned scratches were covered with an adhesive tape (Scotch Crystal Tape 600, 3M, Rüschlikon, Switzerland) to protect the area beneath it from erosion. These reference areas covered parts of the dentine and left a 2 mm window of exposed dentine to be subjected to the erosion attacks.

### Erosive Attacks

The six groups were subjected to four erosive attacks using the following formulas: group 1: Zurich tap water (0.07 ppm fluoride, pH = 7.6, negative control); group 2: 1.2% green tea extract (0.39 ppm fluoride, pH = 5.8,) (OM24, Omnimedica, Schlieren, Switzerland) with tap water; group 3: Red Bull (pH = 3.5) (Red Bull, Baar, Switzerland); group 4: Red Bull Light (pH = 3.5); group 5: Red Bull + green tea extract (pH = 3.7); group 6: Red Bull Light + green tea extract (pH = 3.7). Each group underwent four cycles of erosive attacks and remineralisation. Each erosive attack lasted for 10 min at 25°C with 8 ml of the respective erosive formula, which was pipetted inside prefabricated containers (each two samples in one container) and kept without agitation. Afterwards, the samples were rinsed briefly with deionised water and remineralised with artificial saliva^23^ for 60 min at 37°C.

### Determination of Dentine Wear

Before starting the erosive attacks, baseline profiles were recorded with a stylus profilometer (Perthometer S2, Mahr, Göttingen, Germany). After finishing the erosive procedure (four erosive attacks), final profiles were recorded. A prefabricated jig helped ensuring the exact positioning and repositioning the samples into the profilometer. Five parallel profiles with a distance of 250 µm and an accuracy of 40 nm were recorded for each sample. All profiles were recorded under wet conditions and followed the same recording protocol of an earlier study.^1^ The erosive dentine wear was calculated by superimposition of the baseline profiles with the respective final profiles. For exact superimposition, the unaltered reference areas (protected by the adhesive tape during the erosive attacks) and the two parallel scratches were used. Table 1 summarises the study design.

**Table 1:** Flow diagram detailing the study design

Preparation of 90 dentine samples from 15 bovine incisors 6 samples (A to F) from each incisor					
Allocation of the samples in six groups (n = 15 each)					
Group 1 Samples A	Group 2 Samples B	Group 3 Samples C	Group 4 Samples D	Group 5 Samples E	Group 6 Samples F
Recording of baseline profiles					

Four cycles					
Erosive attack (10 min, 25°C)					
Tap water	Green tea extract 1.2%	Red Bull	Red Bull Light	Red Bull + green tea extract	Red Bull Light + green tea extract
Rinse with deionised water					
Storage in artificial saliva (60 min, 37°C)					
Recording of final profiles					

### Statistical Analysis

Median and interquartile ranges (IQR) of the erosive dentine wear in each group were calculated. The groups were pairwise compared to each other using Wilcoxon-signed rank test and the resulting *P* value was corrected after Holm (α = 0.05). All data was analysed using the statistical program R (The R Foundation for Statistical Computing; Vienna, Austria; www.R-project.org).

## Results

**Fig 1:**
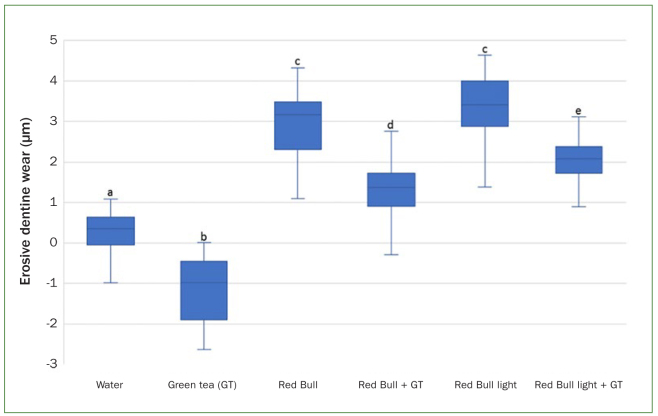
Erosive dentine wear (median + Interquartile range, IQR = whiskers) in the different experimental groups. Same small letters indicate no statistically significant difference.

The median and IQR of the erosive dentine wear in each experimental group was calculated as follows: tap water: 0.4 µm (0.6); green tea extract: –1.0 µm (1.3); Red Bull without green tea supplementation: 3.3 µm (1.0); Red Bull with green tea supplementation: 1.2 µm (0.6); Red Bull Light without green tea supplementation: 3.3 µm (0.9); Red Bull Light with green tea supplementation: 2.0 µm (0.4). Except for the groups treated with Red Bull without green tea supplementation and Red Bull Light without green tea supplementation, all differences between other groups were statistically significantly different (*P* < 0.05). Figure 1 depicts the erosive dentine wear in each group.

## Discussion

Dietary acids are predominant factors in the aetiology of erosive teeth wear. Erosive potential of soft and energy drinks and attempts to reduce it have been investigated in several studies. This *in-vitro* study was carried out to investigate the effect of green tea extract on the erosive potential of a widely consumed energy drink.

Erosive wear was measured on bovine dentine using profilometric analysis in this study. Bovine dentine has already been used in several erosion studies.^5, 21, 26^ In comparison to human teeth, bovine teeth are more readily available, have larger surfaces and allow preparing several samples from the same tooth, and exhibit higher homogeneity of mineral compositions.^34^ The suitability of stylus profilometer to measure erosive tooth loss was also reported in an earlier study.^3^ One shortcoming of this method is the possible interference with the exposed organic collagen network of the eroded dentine, and thus not reflecting the ‘actual’ loss of dentine. However, this is also the case when utilising optical profilometry. Furthermore, samples need to be dry when utilising optic profilometry which might result in dentine desiccation and, again, in profile mis-recordings. The fact that each group was created from 15 dentine samples from each incisor of the 15 incisors used in this study, ensured a certain harmony between the groups. Erosive attacks lasted for 10 min at 25°C. This duration and temperature do not resemble the clinical situation, where soft drinks are consumed at lower temperatures and have shorter contact time with tooth surface. Consuming soft drinks at higher temperatures was found to cause higher erosive dentine and enamel wear. This was attributed to the well-known fact that chemical reactions – in this case between acids in soft drinks and calcium in tooth hard tissue – accelerate with rising temperatures.^33, 36^ However, this helped to investigate the protective effect of green tea under such extreme conditions. Samples were remineralised in – protein-free – artificial saliva after each erosive attack in this study. It was assumed that the presence of proteins in artificial salivas would hamper the remineralisation, and thus might give non-realistic protective effect.^18^ However, other studies found artificial salivas – with or without proteins – to be comparable with each other and with human saliva in in-vitro studies regarding their remineralisation effect, especially when the samples are subjected to more than one erosive attack.^6, 7^ This remineralising step resembles the clinical situation, where dentine gets remineralised with saliva after the erosive attack is over.

In this study, 1.2% supplementation with green tea extract could significantly reduce the erosive dentine wear caused by Red Bull and Red Bull Light. As mentioned earlier, this reduction in the erosive potential is attributed to the presence of EGCG, which inhibits the MMPs and stabilises the dentine matrix. Dentine matrix was found to reduce dentine erosive wear especially when it reaches a certain thickness, as erosive substances must first penetrate it before reaching new ‘sound’ dentine surface and cause further wear.^17^ This inhibition of MMPs to reduce erosive wear was also investigated and proved successful using iron.^21^ Similar results were also noticed in the study of Barbosa et al,^5^ where the same green tea extract used in this study was found to reduce the erosive potential of some soft drinks (Coca Cola, Light Coca Cola, Kuat guarana and Sprite Zero). The green tea extract used in this study contained 30% EGCG and 0.39 ppm fluoride. It is not likely that this small amount of fluoride would play a synergic role in reducing the erosive potential.

As shown in Figure 1, negative final profiles were recorded in the dentine samples treated only with green tea extract. Thus, it could be speculated that a thin film of the green tea extract was precipitated on the surfaces of these samples. It is safe to assume that this film played a synergic protective effect against erosive wear. Nevertheless, it could also be speculated that the less erosive wear measured in the modified-drinks groups might also be attributed to the precipitation of such film. In other words, green tea extract might only have built a precipitation film on the dentine samples in these groups without reducing the erosive potential of the energy drinks per se. In the study by Barbosa et al,^5^ no group was treated only with green tea extract and therefore this possible effect was not mentioned or observed, respectively.

The supplementation using green tea extract did not deeply affect the pH value of the tested energy drinks in this study. The pH value raised only by 0.2 for both drinks (3.5–3.7). This slight raise of the pH value was also reported by Barbosa et al,^5^ where the pH value of most of the tested soft drinks also raised only by 0.1. It is well known that the pH value is one of the relevant factors in the aetiology of erosive wear, and thus this raise of the pH value – even when slight – of the tested drinks might have affected the erosive wear observed in this study.^37^ Furthermore, Barbosa et al^5^ stated that the supplementation of the green tea extract did not alter the taste of the tested soft drinks, which is rather expected by such slight supplementation (1.2%). The taste of other soft drinks was also reported not to be altered after a supplementation of calcium.^4^

## Conclusion

Within the limits of this study, green tea extract could present a valuable reduction in erosive potential of energy drinks *in vitro*. It should be stated that even if the erosive potential of such drinks is kept at minimum or even eliminated, further general health factors associated with the consumption of such drinks should always be kept in mind.
